# DeltaMSI: artificial intelligence-based modeling of microsatellite instability scoring on next-generation sequencing data

**DOI:** 10.1186/s12859-023-05186-3

**Published:** 2023-03-01

**Authors:** Koen Swaerts, Franceska Dedeurwaerdere, Dieter De Smet, Peter De Jaeger, Geert A. Martens

**Affiliations:** 1 Department of Laboratory Medicine, AZ Delta General Hospital, Deltalaan 1, 8800 Roeselare, Belgium; 2 RADar Innovation Center, AZ Delta General Hospital, Roeselare, Belgium; 3 Department of Pathology, AZ Delta General Hospital, Roeselare, Belgium; 4 Department of Biomolecular Medicine, Ghent University, Ghent, Belgium

**Keywords:** Microsatellite instability, Indel length distribution analysis, DNA mismatch repair deficiency, Targeted resequencing, Immunotherapy, Logistic regression, Support vector machine, Machine learning

## Abstract

**Background:**

DNA mismatch repair deficiency (dMMR) testing is crucial for detection of microsatellite unstable (MSI) tumors. MSI is detected by aberrant indel length distributions of microsatellite markers, either by visual inspection of PCR-fragment length profiles or by automated bioinformatic scoring on next-generation sequencing (NGS) data. The former is time-consuming and low-throughput while the latter typically relies on simplified binary scoring of a single parameter of the indel distribution. The purpose of this study was to use machine learning to process the full complexity of indel distributions and integrate it into a robust script for screening of dMMR on small gene panel-based NGS data of clinical tumor samples without paired normal tissue.

**Methods:**

Scikit-learn was used to train 7 models on normalized read depth data of 36 microsatellite loci in a cohort of 133 MMR proficient (pMMR) and 46 dMMR tumor samples, taking loss of MLH1/MSH2/PMS2/MSH6 protein expression as reference method. After selection of the optimal model and microsatellite panel the two top-performing models per locus (logistic regression and support vector machine) were integrated into a novel script (DeltaMSI) for combined prediction of MSI status on 28 marker loci at sample level. Diagnostic performance of DeltaMSI was compared to that of mSINGS, a widely used script for MSI detection on unpaired tumor samples. The robustness of DeltaMSI was evaluated on 1072 unselected, consecutive solid tumor samples in a real-world setting sequenced using capture chemistry, and 116 solid tumor samples sequenced by amplicon chemistry. Likelihood ratios were used to select result intervals with clinical validity.

**Results:**

DeltaMSI achieved higher robustness at equal diagnostic power (AUC = 0.950; 95% CI 0.910–0.975) as compared to mSINGS (AUC = 0.876; 95% CI 0.823–0.918). Its sensitivity of 90% at 100% specificity indicated its clinical potential for high-throughput MSI screening in all tumor types.

*Clinical Trial Number/IRB* B1172020000040, Ethical Committee, AZ Delta General Hospital.

**Supplementary Information:**

The online version contains supplementary material available at 10.1186/s12859-023-05186-3.

## Background

Microsatellites are DNA elements composed of short repetitive motifs that are prone to misalignment and frameshift mutations during cell division [1, 2]. In healthy cells, the ensuing small indels or single-base mispairs caused by polymerase slippage [3] are corrected by heterodimer enzyme complexes of the DNA mismatch repair (MMR) system encoded by MMR genes MLH1, MSH2, PMS2 and MSH6. DNA mismatch repair deficiency (dMMR) is caused by germline or somatic mutations in these MMR genes or by their epigenetic silencing [4, 5]. It results in the progressive accumulation of genetic alterations, potentially dysregulating many oncogenes or tumor suppressor genes. The molecular hallmark of dMMR is microsatellite instability (MSI), with expansions or contractions in the number of tandem repeats throughout the genome. This phenomenon is observed in a considerable proportion of colorectal, endometrial, gastric, pancreatic, brain, biliary tract, urinary tract and ovarian tumors [1, 6].

As dMMR is caused by loss of function variants in the mismatch repair genes, immunohistochemistry (IHC) for loss of MLH1, MSH2, MSH6 and/or PMS2 protein expression is widely used as inexpensive screening test for dMMR [1, 7–11]. An alternative and equivalent approach [9, 11, 12], is the molecular analysis of MSI by detecting shifts in the fragment length (indel) distribution of microsatellite repeats [8, 9, 11, 13, 14]. This is typically done by visual inspection of the fluorescent PCR products of a limited set of microsatellite markers (MSI-PCR, e.g. the Bethesda panel [14]). This requires highly-trained experts and is labour-intensive. The rapidly increasing number of indications for MSI screening since the approval of PD-1 inhibitors in all microsatellite unstable solid cancers [15] thus creates pressure on labs that rely on classical MSI-PCR. A fast and cost-efficient alternative is to embed MSI analysis in bioinformatic pipelines applied on next-generation sequencing (NGS) data, that are increasingly being collected as standard-of-care diagnostic workup in many dMMR-prone cancer types.

Bioinformatic tools for MSI detection on NGS data are available in different flavours: (A) by comparing indel distributions of microsatellites in paired tumor-normal samples, on exome data (MSIsensor, MANTIS, MSIseq) or targeted gene panels (USCI-msi) [16–18], (B) by comparing indel distributions in a tumor sample versus a fixed reference set on targeted gene panels (mSINGS, MSIFinder) [19, 20] or (C) by analysing the number of single nucleotide variants and indels throughout the genome to detect the hypermutated status as consequence of dMRR. (MSIseq) [21]. An overview of these tools and their features is presented in Table 1.

**Table 1:** Overview of published MSI screening scripts on NGS data

Tool	Year	Normal required	Data type	Cohort size	Tumor type	Number of MSI loci	Loci selection	Method	Raw data	References
MSIsensor	2014	Yes	Exome/large panels	242	Endometrium	> 1000	Using all	Peak based, chi-square test	Bam	10.1093/bioinformatics/btt755
MSIseq	2015	Yes	Exome	526	4 types			Variant based, decision tree	Bam	10.1038/srep13321
Mantis	2016	Yes	Exome	458	6 types	> 100	Using all	Peak based, distance score	Bam	10.18632/oncotarget.13918
MSIpred	2018		Exome	1432			–	Variant based, support vector machine	Maf	10.1038/s41598-018-35682-z
MSI-ColonCore	2017	Yes	Capture	91	Colorectal	22	Manual	Peak based, based on MSIsensor	Bam	10.1016/j.jmoldx,2017.11.007
USCI-msi	2020	Yes	Capture	64	Colorectal	9	Automatic	Location selection optimisation, uses Mantis	Bam	10.1186/s12967-020-02373-1
NovoPM-MSI	2020	Yes	Capture	113	Colorectal	19	Manual	Peak based, Man-Witney U-test	Bam	10.3892/ol.2020.11702
MIAmS	2019	No	Amplicon	286		6	Manual	Peak based, mSINGS + support vector machine correction	Fastq	10.1093/bioinformatics/btz797
MSIsensor-pro	2020	No	Exome	1532	Colorectal	11,666	Automatic	Peak based, multinomial distribution	Bam	10.1016/j.gpb.2020.02.001
MSIFinder	2021	No	Capture	419		54	Manual	Peak based, random forest	Bam	10.1186/s12859-021-03986-z
MEM	2021	No	Capture	146	Colorectal	5	Manual	Peak based, expectation–maximisation	Fastq	10.3390/cancers13164203
mSINGS	2014	No	Capture	324	Colorectal	15-2957	Manual	Peak based, Z-test	Samtools mpileup	10.1373/clinchem.2014.223677
DeltaMSI		No	Capture/amplicon	331	11 types	28	Automatic	Peak based, logistic regression and support vector machine	Bam	Present report

Overview of MSI screening scripts with year of initial publication and reference. Defining features of these scripts are the type of NGS data used (small gene panels to exomes), the number of microsatellite loci used, the metrics used to score indel distributions as stable/unstable and manual versus automatic selection of loci with diagnostic power. The table also lists the number of tumor samples used for training and validation and the number of tumor types examined

Microsatellite instability is a continuous feature that becomes more marked with increasing number of cell divisions of MMR deficient cells. Also, some microsatellite loci appear more prone to instability in specific tumor types and MSI loci optimized for colorectal cancers might perform sub optimally in endometrial and other cancer types [22]. The sensitivity of molecular MSI screening can be improved by sequencing paired tumor-normal tissue and by the use of exomes or very large genomic panels. One such assay, the FDA-approved MSIsensor script on MSK-Impact large-panel capture data of tumor-normal pairs, outperformed conventional MSI-PCR in both colorectal and endometrial carcinoma [23]. However, the cost of such assay is prohibitive and in most clinical settings, sequencing of solid tumors is restricted to small NGS panels.

When applied on a limited set of MSI markers in small gene panels, commonly used scripts such as mSINGS [19] and MSIFinder [20] have been shown to achieve similar diagnostic performance as classical Bethesda-compliant PCR, at least in colon cancer. In endometrial cancers, mSINGS was still inferior to IHC, mainly because it failed to detect the minimal shifts in microsatellite indel distributions that occur in non-colorectal tumors. One possible explanation is that mSINGS, and other widely used scripts such as MSIFinder, rely on a single parameter—the number of discrete, integrated peaks in an indel distribution—for binary scoring at locus level. This simplification can result in inaccurate MSI screening in case of left-shifted distributions or minimal microsatellite shifts, as illustrated in Additional files (Additional file 2: Figure S1) and [12].

Therefore, the aim of this study was to develop a script, DeltaMSI, that can process the full complexity of indel distribution (area under the curve, length of major peak and number of peaks). Its novel and defining feature versus existing scripts is that it uses the raw aligned read data (normalized read depth per position) of microsatellite loci as input parameter, leveraging the power of machine learning with scikit-learn [24] to handle the associated data complexity. To allow its clinical use and ease of implementation, additional (but not necessarily novel) required features were: (1) applicability on small NGS panels (unlike MSIsensor, MSIseq, Mantis that require exome data); (2) not dependent on a fixed baseline reference set of healthy tissue or MSS tumors (unlike mSINGS); (3) automatic selection of optimal loci (like MSIsensor-pro) and (4) not dependent on tumor-normal paired sequencing (unlike MSIsensor, MSIseq, MSIpred and others).

All published tools for MSI screening on NGS data report high diagnostic performance versus MSI-PCR as reference. However, as shown by Dedeurwaerdere et al. all dMMR detection techniques based solely on molecular methods fail to detect up to 5–10% of dMMR tumors with demonstrated loss of MLH1/MSH2/PMS2 or MSH6 immunohistochemical staining, either for biological reasons (minimal MSI shifts) or sensitivity issues (low tumor cell percentage). To assess real-world clinical performance, DeltaMSI was therefore trained and validated on samples classified by IHC as independent, non-molecular method for clinical truth. The resulting DeltaMSI script showed high robustness during implementation and satisfactory diagnostic performance in a large real-world data set of solid tumors of various origin tissues.

## Implementation

### Study specimens

All FFPE biopsy specimens were obtained from cancer patients as part of standard clinical care at AZ Delta General Hospital from Jan 1, 2019 to Dec 31, 2021. Training and validation was done on a cohort consisting of 1072 unselected, consecutive solid tumor samples (large variety of origin tissues) processed for diagnostic NGS by panel-based capture assay and included a subset of 215 samples with IHC results. A second validation was done on a cohort of 116 consecutive samples processed by IHC and sequenced by an amplicon-based library preparation chemistry of the same gene panel design.

### Wet lab pre-processing

Immunohistochemistry (IHC) for MSH2, MSH6, PMS2 and MLH1 was performed on 5-µm thick sections of formalin-fixed, paraffin-embedded (FFPE) tumor tissue on a Ventana, Benchmark Ultra device (Ventana Medical Systems, Arizona, USA) as described [12]. Tumors were classified as dMMR if no nuclear staining or nuclear staining in less than 10% of invasive tumor cells for 1 or several markers was seen in the presence of a positive internal control (inflammatory and stromal cells). Tumors with nuclear staining for all for markers in at least 10% of invasive tumor cells were classified as pMMR. DNA was extracted from 10-µm thick sections of the same FFPE tissue blocks as used for immunohistochemistry, using the Cobas DNA Sample Preparation Kit (Roche, Basel, Switzerland) with macrodissection guided by haematoxylin eosin staining, with elution in 10 mM Tris–HCl pH8.0. NGS was performed on an Illumina MiSeq (PE150) using a custom 138 kb (36 genes) hybridization capture-based gene panel (NimbleGen SeqCap EZ HyperPlus, Roche) gene panel that additionally included 36 microsatellite loci [12] (Additional file 2: Table S1): 15 proposed by Salipante [19], 7 from the Idylla assay (Biocartis, Belgium) [25], 10 proposed by Hause as informative for non-colorectal cancers [22] and 4 regions from the updated Bethesda guideline [14]. DeltaMSI was additionally implemented and validated on samples sequenced using an amplicon-based method (AmpliSeq for Illumina Focus Panel, Illumina) customized with these same 36 microsatellite loci. Both NGS methods achieved a limit of detection of 1% variant allele frequency for SNV, at minimal unique read depth of 500x.

### Data pre-processing

Reads were aligned to the human reference genome (hg19) with BWA-mem2 (2.2.1). Sorting and duplicate marking were performed using elPrep (5.0.2) [26] and SAMtools (1.13) [27] for the indexing of the bam files. To simplify the use of our method, read filtering was integrated in the proposed DeltaMSI script. Bam files were read by DeltaMSI. Marked duplicates were ignored, and reads were filtered on a mapping quality of at least 20. Only reads overlapping the complete microsatellite regions with a flanking region of 5 bases on each side were used. Regions within samples were filtered with a minimum depth of 30x (similar as mSINGS). The length distribution graphs, obtained by counting the observed fragment lengths, were cleaned by removing lengths with a depth of only 1 read (to decrease the complexity of the regions). As raw data for modeling the normalized read depth per position for each marker locus was used, obtained by dividing the absolute read number per position by the maximally observed depth of all lengths within that locus, obtaining a value between 0 and 1 for each possible length within that region.

### Machine learning modelling by train-validation-test split approach

**Fig. 1:**
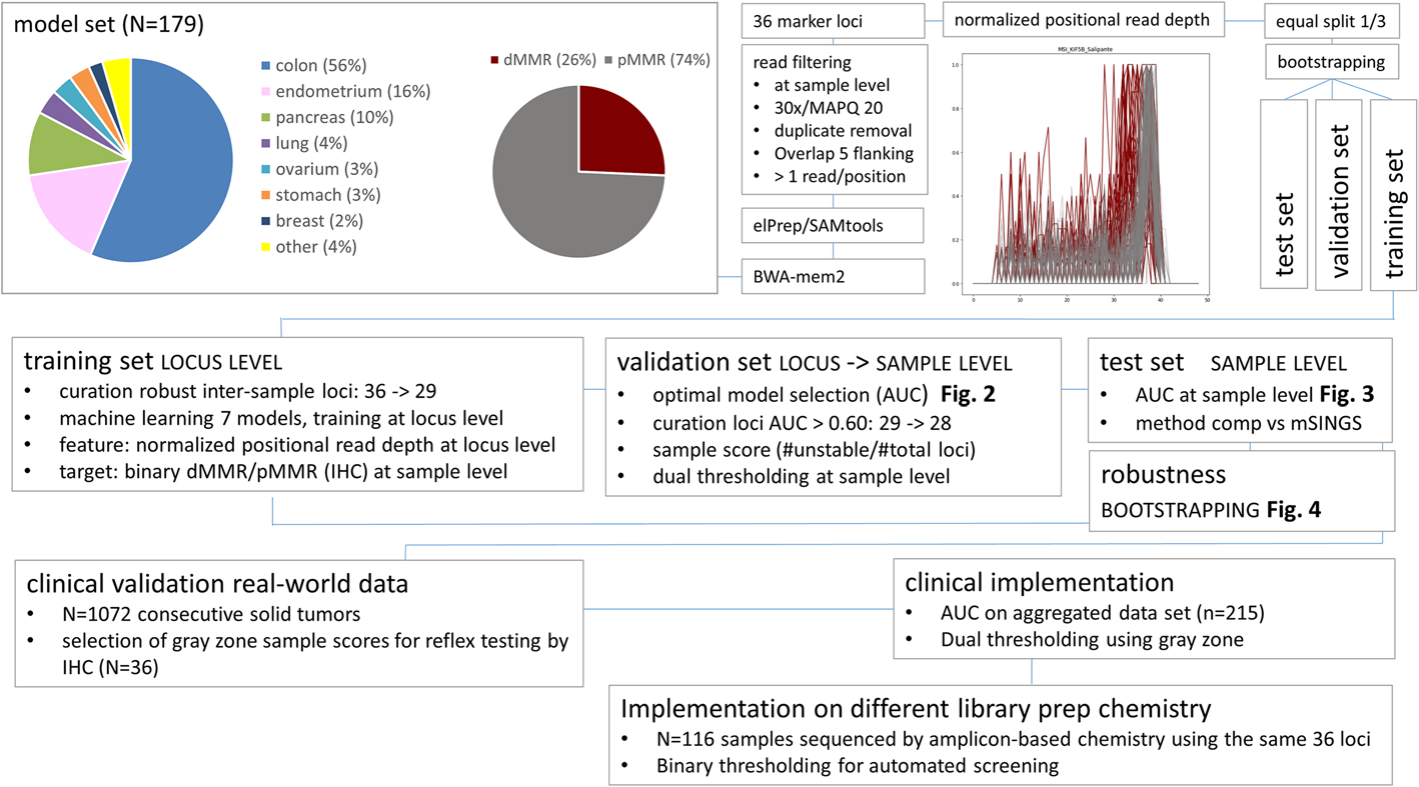
Study design and implementation. The flow chart graphically summarizes the study design, modeling, implementation and clinical validation as described in detail in the Implementation section with reference to the cited figures

**Fig. 2:**
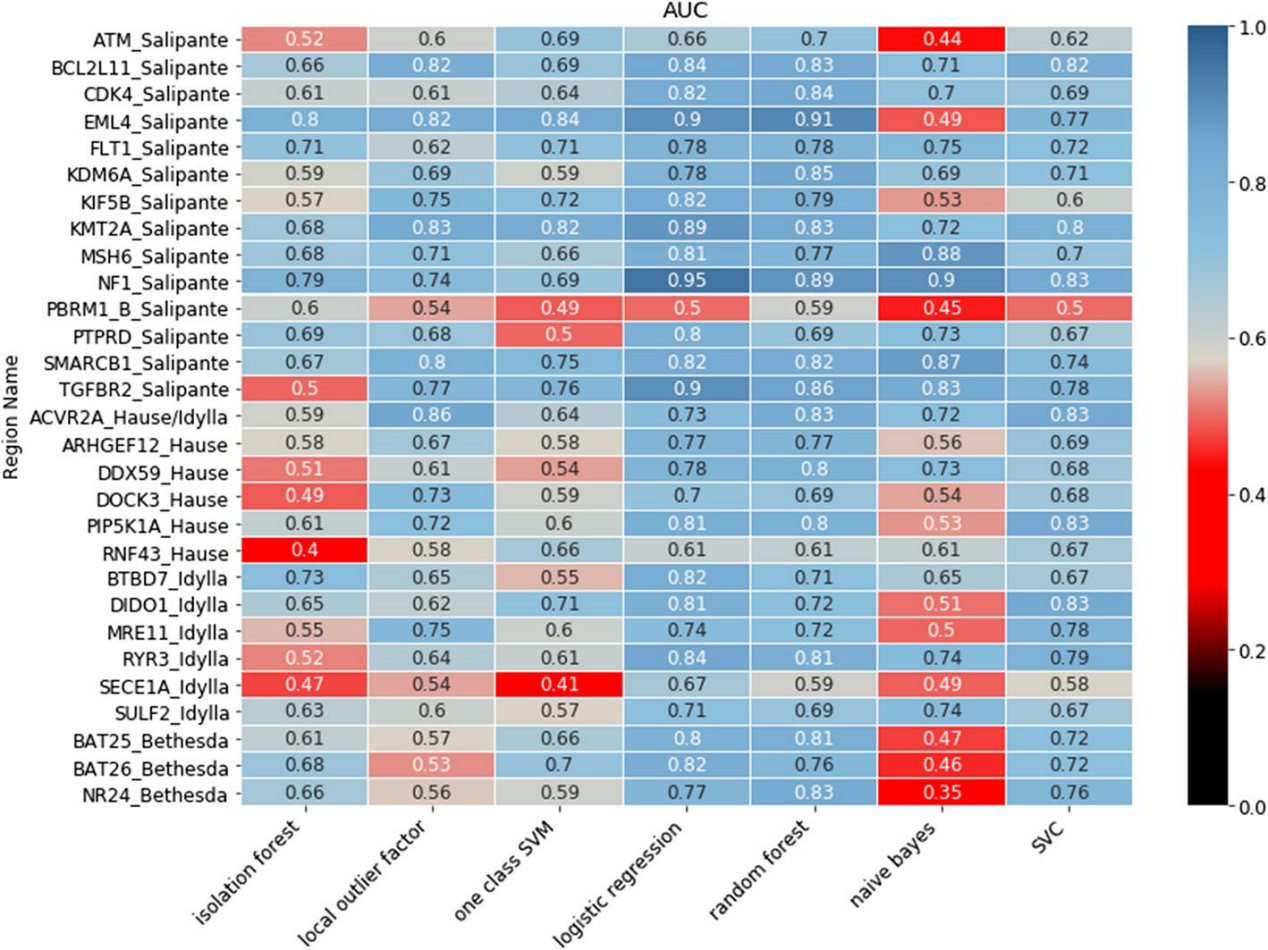
Diagnostic power of 29 individual microsatellite marker loci in the various models to predict dMMR status. 29 of 36 marker loci (Y-axis) showed acceptable coverage within and between samples and were used for machine learning by the indicated models (X-axis). Marker loci are denoted by gene name (capitals) and source (Salipante/Idylla®assay/Hause/Bethesda panel) with full genomic coordinates in Additional file 2: Table S1. Models were trained versus outcome at sample level by IHC (dMMR/pMMR) assuming all loci of a sample classified by IHC as pMMR to be stable, and all loci of a sample classified as dMMR to be unstable. Models included isolation forest, local outlier factor, one-class support vector machine (SVM), logistic regression, random forest, naive Bayes and support vector classifier (SVC). The heat map shows representative AUC at locus level in the validation set to predict dMMR status at sample level (low to high AUC from red to blue). 28 of 29 loci (with exception of MSI_PBMR1_Salipante) achieved acceptable AUC and were retained in the final DeltaMSI script. Logistic regression and SVC consistently achieved highest AUC and were integrated into the combined voting model of DeltaMSI

Machine learning was done at locus level by scikit-learn (1.1.2) [24] taking normalized read depth per position as feature versus binary dMMR status measured by IHC as target on a model set of 179 samples sequenced by capture NGS (N = 133 pMMR and N = 46 dMMR deficient samples). The model set was randomly split in a training set, validation set and test set of equal size (graphical study design in Fig. 1). The training set was first used to select the subset of loci with good raw data quality based on acceptable coverage within sample and in 75% of all training set samples: 29 of 36 loci were retained (Fig. 2). Scikit-learn was used to train and compare seven machine learning models. The methods of machine learning were selected considering the small sample size, excluding neural networks, and the low prevalence of dMMR samples in diagnostic settings, focussing on outlier methods and methods able to handle unbalanced datasets.

Three outlier methods (isolation forest, local outlier factor and one class support vector machine) were trained with pMMR samples only. The other methods (logistic regression, random forest, naive Bayes and support vector classifier (SVC)) were trained with an unbalanced set of 45 pMMR and 16 dMMR samples. For each method hyperparameter tuning was performed using a grid search with a kfold of 3. To allow unbiased comparison, each model used the same kfold split of the samples, regardless of the number of filtered samples for that region within that set.

Next, the validation set was used to select the best performing models and loci based on the AUC score at locus level. The highest AUC were obtained with logistic regression, SVC and random forest (Fig. 2). Random forest was removed due to strong overfitting of each region. 28 of 29 marker loci achieved AUC of 0.60 or higher in both logistic regression and SVC. The latter two models were selected for the final modeling and additionally integrated into a combined voting model, hence DeltaMSI, that scores regions as unstable if both models predict the region as unstable. For each model, the percentage of loci scored as unstable was calculated (sample score). This sample score was then used to explore optimal diagnostic thresholds for dMMR/pMMR classification at sample level in the test set.

ROC analysis was done by MedCalc (version 19.2.1, MedCalc software Ltd, Mariakerke, Belgium) and Python scikit-learn [24]. A method comparison was done between DeltaMSI and mSINGS v4.0.

## Results

### Clinical validation real world data

**Fig. 3:**
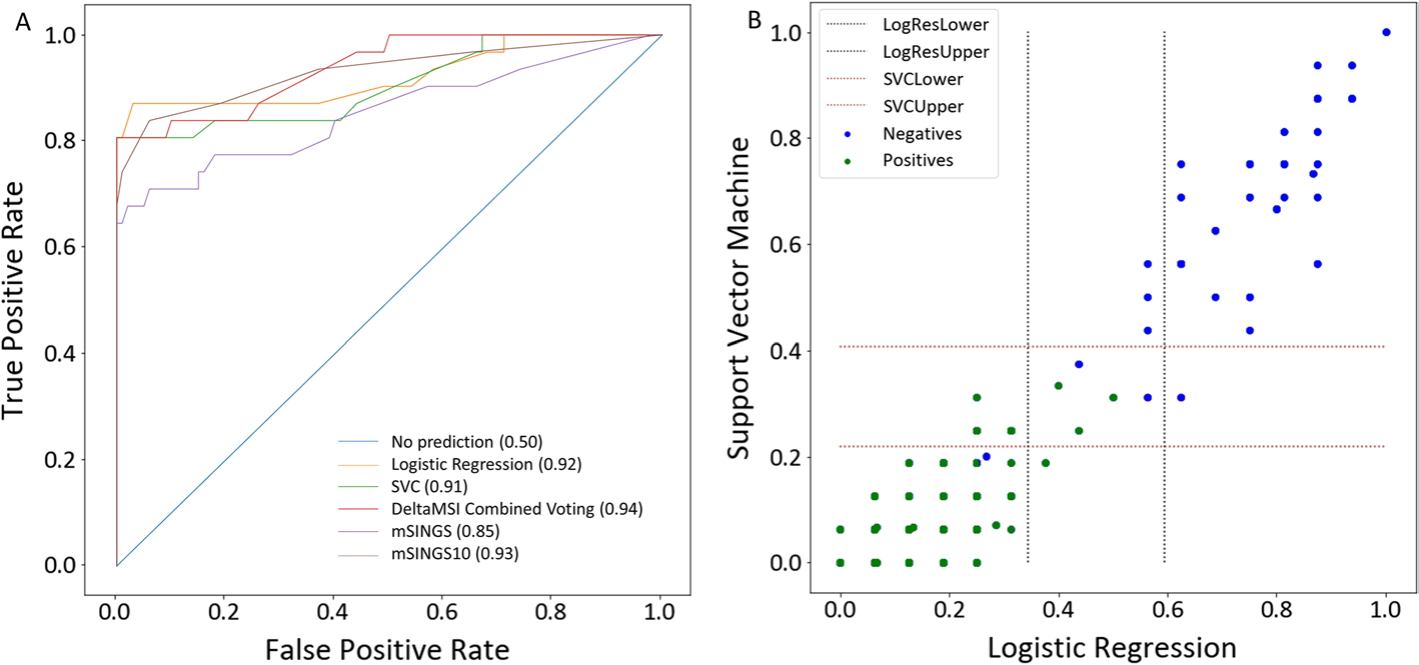
Diagnostic power of DeltaMSI to predict dMMR status at sample level. **A** representative AUC of logistic regression, SVC and the combined voting model (DeltaMSI) on 28 marker loci in the test set in one of 50 representative bootstrap simulations, as compared to mSINGS on these same 28 loci and same bootstrap simulations (mSINGS) and an optimized, previously published [12] mSINGS done on 10 top-performing marker regions with a fixed optimized baseline set (mSINGS10). **B** Concordance of classification at sample level (blue dots, dMMR, IHC positive for loss MLH1/MSH2/PMS2 or MSH6 expression; green dots, pMMR, IHC negative) by logistic regression and support vector classifier and provisional dual thresholding with gray zone interval for subsequent clinical validation on real-world data

The diagnostic power of logistic regression, SVC and the combined voting model (hence DeltaMSI) were evaluated in the test set, in comparison to mSINGS (v4.0). Two implementations of mSINGS were evaluated. First, a previously published implementation [12] that has been in clinical routine use in our centre since 4 years. It makes use of an optimized fixed reference set of 15 pMMR colorectal cancer samples and 10 of 15 loci original Salipante loci [19] in the capture panel design (hence mSINGS10). Second, an out-of-the-box implementation on all 28 marker loci of DeltaMSI, using the randomly selected pMMR samples of the train-validation set that was used in the training of DeltaMSI as reference set (hence mSINGS). ROC analysis (Fig. 3A) indicated strong diagnostic power of DeltaMSI, with AUC of logistic regression, support vector classifier and the combined voting model of 0.92, 0.91 and 0.94, respectively, similar to mSINGS10 (AUC = 0.93) but superior to mSINGS (AUC = 0.85).

The test set was also used to optimize cutoffs for subsequent clinical verification on real-world data. First, dual thresholding was tested, with a lower cutoff below which samples are pMMR with 95% specificity and a higher cutoff above which samples are dMMR with 95% specificity (Fig. 3B) and separated by a gray zone result interval. This thresholding was then evaluated in a real-world data set of 1072 consecutive, unselected samples of solid tumors, in comparison to mSINGS on the same 28 marker loci, using 0.20–0.30 as gray zone for mSINGS. The data set included solid tumors of a wide variety of origin tissues, including colorectal (20%), lung (27%), breast (14%), pancreas (6%), ovarium (5%), melanoma (5%), endometrium (4%), brain (4%), prostate (2%), GIST (1%) and stomach (1%) cancers (Additional file 2: Table S2). On the colorectal cancers (N = 214), DeltaMSI and mSINGS with dual thresholding achieved only a moderate concordance of MSS/MSI classification (72%). The estimated prevalence of MSI (21% and 18% for DeltaMSI and mSINGS respectively) was higher than the 10%-15% epidemiologically expected in colorectal cancers [22], suggesting suboptimal thresholding. Therefore, an additional set of N = 36 samples was selected within the gray zone result interval of DeltaMSI for independent verification by IHC and aggregated these into our initial model set for iterated ROC analysis on a total of N = 215 samples.

In these 215 samples, DeltaMSI achieved excellent AUC of 0.95 (95% CI 0.91–0.98), similar to the optimized mSINGS10 (AUC = 0.93, 95% CI 0.89–0.96) but statistically superior to mSINGS on all 28 marker regions (AUC = 0.876, 95% CI 0.82–0.92, *P* < 0.05) (Table 2). A single binary threshold could be defined for DeltaMSI (sample score > 0.26) above which microsatellite instability was predicted at > 98% specificity and likelihood ratios above 70 (illustrated in Additional file 2: Table S2). This corresponded to a sensitivity of 90% (95% CI 76–96%). Further decreasing binary cutoff had no impact on exclusion of MSI (negative likelihood ratio) and thus no beneficial impact on screening rate: in total 5 cases were missed of which 2 for obvious technical reasons (low tumor cell percentage < 40%).

**Table 2:** AUC of DeltaMSI models and mSINGS versus IHC outcome Table shows the AUC (95% CI) of models tested in DeltaMSI development (logistic regression, SVC and the combined voting) as compared to mSINGS on all 28 marker regions (mSINGS) and mSINGS on 10 top-performing regions (mSINGS10) in N = 215 samples confirmed by IHC (179 of the original model set plus 36 Gy zone result samples that were additionally tested by IHC)

Variable	AUC	95% CI	P	Cut-off	Sensitivity	95% CI	Specificity	95% CI	+LR	−LR
Logistic regression	0.949	0.909–0.975	0.0216	0.26	88	75.7–95.5	98.79	95.7–99.9	72.6	0.12
SVC	0.950	0.911–0.976	0.0198	0.26	90	78.2–96.7	98.79	95.7–99.9	74.25	0.1
Combined Voting	0.950	0.910–0.975	0.0224	0.26	88	75.7–95.5	100	97.8–100.0	∞	0.12
mSINGS10	0.931	0.887–0.962	0.0295	0.26	90	78.2–96.7	98.79	95.7–99.9	74.25	0.1
mSINGS_allregions	0.876	0.823 to 0.918		0.29	68	53.3–80.5	98.17	94.7–99.6	37.17	0.33

P value versus mSINGS_allregions. Table also indicates the proposed binary cut-off for clinical use and the associated sensitivity/specificity and positive (+LR) and negative (−LR) likelihood ratios to predict or rule out dMMR status

### Robustness of DeltaMSI

**Fig. 4:**
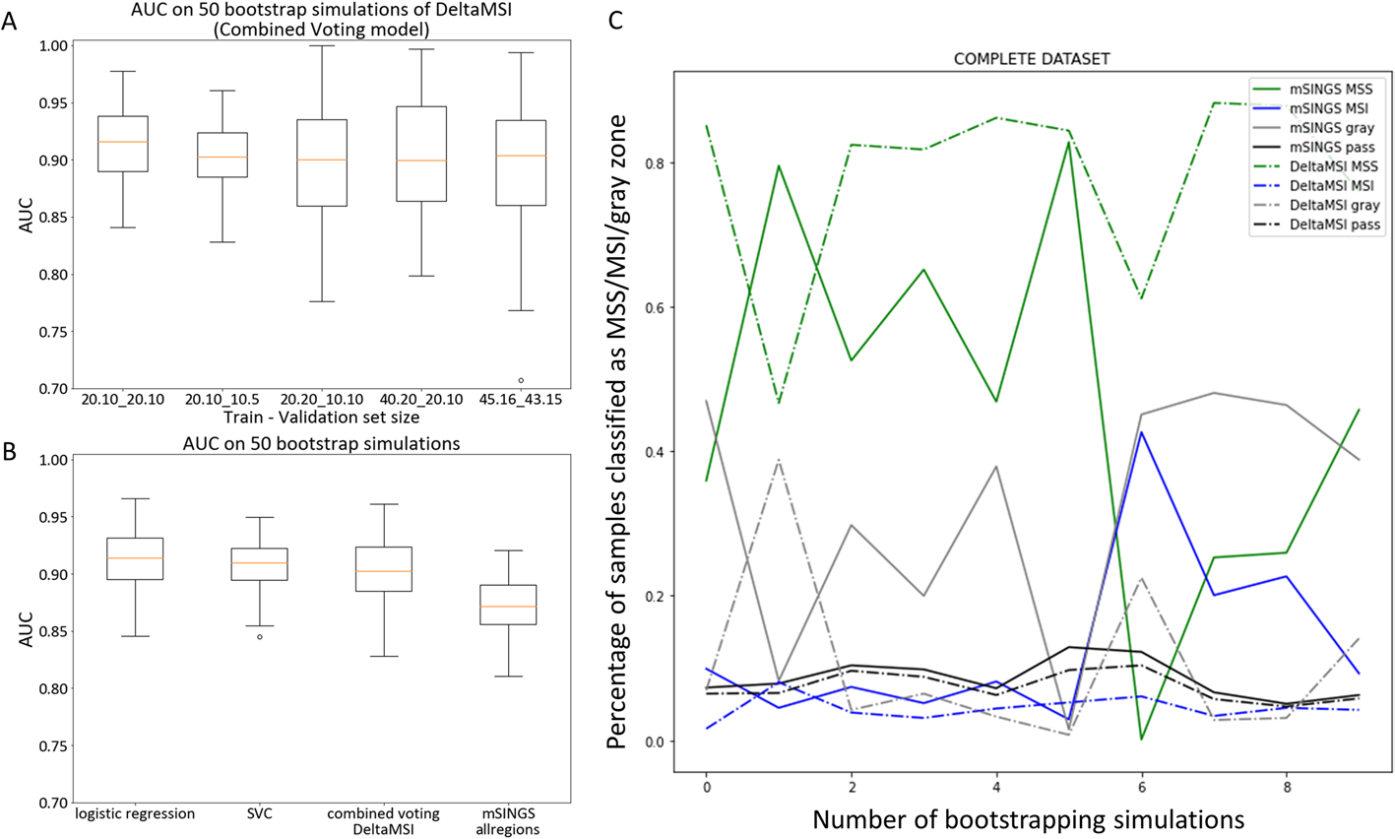
Robustness of DeltaMSI as compared to mSINGS. **A** Whisker plots show AUC (Q25-median-Q75) of the DeltaMSI Combined voting model for the indicated sample sizes (X-axis) in the train and validation sets over 50 bootstrap simulations. The AUC in the smallest model set (20.10_10.5, indicating 20 pMMR.10dMMR samples in the training set_10 pMMR.5dMMR samples in the validation set) was comparable to the larger set used for initial training of the model (45.16_43.15). **B** AUC over 50 bootstrap simulations for the smallest sample size (20.10_10.5) of logistic regression (logres), SVC and the combined voting model (DeltaMSI) as compared to mSINGS on the same 28 regions and same bootstrap samplings. **C** robustness over 10 bootstrap simulations using the minimal sample size (20.10_10.5) in a real-world data set of N = 1072 consecutive solid tumor samples. Plots indicate the percentage of samples classified as MSS/pMMR (green lines) or MSI/dMMR (blue lines) or gray zone by DeltaMSI (dotted lines) or mSINGS (solid lines)

DeltaMSI was developed on a relatively well powered sample set, which is not always available in routine diagnostic settings. To evaluate the impact of sample size on AUC, bootstrapping simulations were performed on training/validation sets of decreasing size. 50 simulations were run on 5 sample sizes: 20-10:20-10; 20-10:10-5; 20-20:10-10; 40-20:20-10; 45-16:43-15 (train pMMR-dMMR: validation pMMR-dMMR). Decreasing sample size down to the smallest sample size (20-10:10-5) did not negatively affect AUC (AUC mean: 0.904, median: 0.902, 95% CI 0.873–0.947) as compared to the original sample size (45-16:43-15) (AUC mean: 0.893, median: 0.904, 95% CI 0.782–0.966) for the combined voting model (Fig. 4A) (*P* = 0.25). Comparison of logistic regression, SVC and DeltaMSI (combined voting) versus mSINGS on all 28 regions, in 50 bootstrapping simulations using the smallest 20-10:10-5 train-validation size, confirmed the trend towards superior AUC of DeltaMSI (AUC mean 0.904, median: 0.902, 95% CI 0.873–0.947) as compared to mSINGS (AUC mean: 0.872, median: 0.872, 95% CI 0.831–0.915) (*P* < 0.001) (Fig. 4B). The robustness of mSINGS versus DeltaMSI was also evaluated on the complete real-world data set (n = 1072), using minimal sample size (training 20-10: validation 10-5 pMMR-dMMR): over 10 bootstrapping simulations, DeltaMSI had a more stable performance with less variation across simulations in the percentage of samples classified as MSI/gray zone/MSS classification (Fig. 4C). This indicates that DeltaMSI displays a much lower dependency on the optimal training/validation set than mSINGS.

As additional test for implementation robustness, DeltaMSI and mSINGS were compared on an independent data set 116 (N = 86 pMMR, N = 30 dMMR) solid tumors sequenced using a different library preparation chemistry (amplicon-based), using the exact same 28 loci in the panel design. This cohort included cancers originating from colorectal (56%), endometrium (24%), pancreas (10%), stomach (8%) and other (2%) organs. 50 simulations were performed using 20-10:10-5 train-validation size, and compared to mSINGS. In each simulation the train-validation set was randomly constructed, using the selected pMMR samples for train-validation in DeltaMSI as mSINGS baseline. On amplicon data, both DeltaMSI (AUC mean: 0.98, median: 0.98, 95% CI 0.980–0.985) and mSINGS (AUC mean: 0.97, median: 0.97, 95% CI 0.970–0.972) achieved excellent AUC, but AUC of DeltaMSI was again statistically superior (*P* < 0.001). In total, 4 of 116 (3%) samples (all non-colorectal) showed discrepant results between IHC and molecular methods. One endometrial adenocarcinoma was pMMR on IHC but convincingly MSI by both DeltaMSI and mSINGS, and thus likely indicates a false negative IHC. Of the three samples scored as dMMR by IHC, two samples, a duodenal adenocarcinoma with 60% tumor cells and an endometrial carcinoma with only 20% tumor cells were scored as MSS by both DeltaMSI and mSINGS and one sample, an endometrial carcinoma with only 30% tumor cells, was correctly scored as MSI by DeltaMSI but not mSINGS.

### Standalone tool: novelty and complexity

The novelty of DeltaMSI as compared to previously published MSI calling tools stems from a combination of following features (Table 1): (1) multiparametric analysis of indel distribution shifts instead of simplification to just one parameter such as number of discrete indel peak lenghts; (2) automatic selection of MSI loci with highest diagnostic power in any custom gene panel: such automatic selection was previously described in a few MSI calling tools (USCI-msi, MSIsensor-pro) but in DeltaMSI loci selection and model building are integrated in one command; (3) the combined use of multiple machine learning models on multiparametric indel data, yielding one integrated sample score. Some other MSI calling tools also used machine learning, but these typically relied on a single model and monoparametric data. From a methodological perspective, the use of IHC as outcome for clinical truth benefits clinical robustness of DeltaMSI. To our knowledge, only 3 other tools (Table 1) used IHC outcome, with concordances varying from 80% for MSIFinder [20], 84.3% for USCI-msi [17], up to 92% for MSI-ColonCore [28] (Table 1). The performance of DeltaMSI and mSINGS was tested with multiple bootstraps with a concordance of above 90% and 98% for DeltaMSI versus 82% and 97% for mSINGS on capture and amplicon data, respectively.

DeltaMSI was developed as a standalone tool with easy implementation. The implementation in pseudocode can be found in the Additional file 1. Machine learning today is still hesitantly adopted in routine clinical diagnostic practice, mainly because of opacity of the data handling. To decrease complexity, DeltaMSI creates visual plots (examples in Additional files) of all included loci, that graphically display indel shifts versus all pMMR samples in the train set.

### Compute performance of DeltaMSI

DeltaMSI and mSINGS are both developed in Python and are single threaded. mSINGS is RAM friendly, using only up to 40 MB, while DeltaMSI used up to 6 GB of RAM for both training as predicting the samples (for 28 loci). On a 3Ghz cpu (Intel Xeon Gold) with 256 GB RAM, the creation of the mSINGS baseline with 30 pMMR samples took around 12 min and 30 s (mean over all simulations). The creation of the DeltaMSI model with 30 pMMR and 15 dMMR samples took 11 min and 52 s (mean over all simulations). The prediction by mSINGS takes 26 s, for DeltaMSI it was 10 s.

## Conclusions

DeltaMSI achieved a clinically acceptable sensitivity around 90% at excellent specificity for MSI screening in all common cancer types. Its diagnostic performance was at least as good as that of another widely used script, mSINGS. As compared to mSINGS, however, it offered a higher robustness during implementation, with a diagnostic performance that was less influenced by the composition of the training/baseline samples sets, and thus guaranteeing a more streamlined implementation with less validation efforts. Present study shows that machine learning on complex raw indel distribution data can achieve higher robustness at sample level than scripts running on monoparametric decomplexified data. Molecular screening of MSI in non-colorectal tumor samples, however, remains challenging and the diagnostic gain by DeltaMSI versus mSINGS are limited. Future research should investigate if progressively increasing the gene panel size and the number of microsatellite loci can improve the sensitivity of DeltaMSI and similar scripts.

### Availability and requirements

Project name: DeltaMSI

Project home page: https://github.com/RADar-AZDelta/DeltaMSI

Operating system(s): Platform independent

Programming language: Python

Other requirements: available in Pipfile

License: GPL v3.0

Any restrictions to use by non-academics: See License

Raw sequencing data available on reasonable request to koen.swaerts@azdelta.be.

## Supplementary Information

**Additional file 1.** Implementation pseudocode DeltaMSI.

**Additional file 2.** Supplementary information.

## Data Availability

The datasets used and/or analysed during the current study available from the corresponding author on reasonable request.

## Abbreviations

AUC: Area under the curve of receiver operating characteristics curve
dMMR: DNA mismatch repair deficiency
IHC: Immunohistochemistry
Logres: Logistic regression
MSI: Microsatellite instability
NGS: Next-generation sequencing
SVC: Support vector classifier
