# Supplementary material for: DeltaMSI: artificial intelligence-based modeling of microsatellite instability scoring on next-generation sequencing data
Source: BMC Bioinformatics. 2023 Mar 1;24:73. doi: 10.1186/s12859-023-05186-3 (PMC9976396; doi:10.1186/s12859-023-05186-3)
Supplement: Supplementary file 2 — Additional file 2. Supplementary information. [file 12859_2023_5186_MOESM2_ESM.docx]

**Supplementary information**

**DeltaMSI: Artificial Intelligence-based modeling of microsatellite instability scoring on next-generation sequencing data**

**Koen Swaerts** ^1,2^**, Franceska Dedeurwaerdere** ^3^**, Dieter De Smet** ^1,2^**, Peter De Jaeger**  ^2^ **and Geert A. Martens** ^1,2,4^

^1^ Department of Laboratory Medicine, AZ Delta General Hospital, Roeselare, Belgium; ^2^ RADar innovation center, AZ Delta General Hospital, Roeselare, Belgium; ^3^ Department of Pathology, AZ Delta General Hospital, Roeselare, Belgium; ^4^ Department of Biomolecular Medicine, Ghent University, Gent, Belgium

**1. Genomic coordinates of 36 microsatellite marker loci**

| Chromosome | Start | End | Gene Name | Marker source |
| --- | --- | --- | --- | --- |
| **chr1** | **151196693** | **151196722** | **PIP5K1A** | **Hause** |
| **chr1** | **200594035** | **200594053** | **DDX59** | **Hause** |
| **chr2** | **42499212** | **42499241** | **EML4** | **Salipante** |
| **chr2** | **47641554** | **47641591** | **BAT26** | **Bethesda** |
| **chr2** | **48032735** | **48032758** | **MSH6** | **Salipante** |
| **chr2** | **95849356** | **95849389** | **NR24** | **Bethesda** |
| **chr2** | **111886175** | **111886203** | **BCL2L11** | **Salipante** |
| **chr2** | **148683678** | **148683700** | **ACVR2A** | **Hause, Idylla** |
| **chr3** | **30691861** | **30691886** | **TGFBR2** | **Salipante** |
| **chr3** | **51417597** | **51417615** | **DOCK3** | **Hause** |
| chr3 | 52621581 | 52621611 | PBRM1_A | Salipante |
| **chr3** | **52621904** | **52621929** | **PBRM1_B** | **Salipante** |
| **chr4** | **55598206** | **55598241** | **BAT25** | **Bethesda** |
| **chr4** | **83785558** | **83785578** | **SEC31A** | **Idylla** |
| chr7 | 74608735 | 74608758 | GTF2IP1 | Hause |
| chr8 | 7346860 | 7346909 | DEFB105B | Hause |
| chr8 | 7679722 | 7679741 | DEFB105A | Hause |
| **chr9** | **8341275** | **8341297** | **PTPRD** | **Salipante** |
| **chr10** | **32316536** | **32316564** | **KIF5B** | **Salipante** |
| **chr11** | **94212925** | **94212946** | **MRE11A** | **Idylla** |
| **chr11** | **108188261** | **108188284** | **ATM** | **Salipante** |
| **chr11** | **118353032** | **118353058** | **KMT2A** | **Salipante** |
| **chr11** | **120350631** | **120350657** | **ARHGEF12** | **Hause** |
| **chr12** | **58141999** | **58142023** | **CDK4** | **Salipante** |
| **chr13** | **28942835** | **28942863** | **FLT1** | **Salipante** |
| chr14 | 23652341 | 23652372 | NR21 | Bethesda |
| **chr14** | **93708025** | **93708045** | **BTBD7** | **Idylla** |
| **chr15** | **34157535** | **34157556** | **RYR3** | **Idylla** |
| chr16 | 9934665 | 9934707 | GRIN2A | Salipante |
| chr16 | 14983086 | 14983105 | NOMO1 | Hause |
| **chr17** | **29508814** | **29508840** | **NF1** | **Salipante** |
| **chr17** | **56435155** | **56435175** | **RNF43** | **Hause** |
| **chr20** | **46286315** | **46286337** | **SULF2** | **Idylla** |
| **chr20** | **61536686** | **61536707** | **DIDO1** | **Idylla** |
| **chr22** | **24135963** | **24135990** | **SMARCB1** | **Salipante** |
| **chrX** | **44935872** | **44935895** | **KDM6A** | **Salipante** |

**Supplementary Table 1: genomic coordinates of the 36 microsatellite marker loci used for training.** Name includes MSI_GENE_SOURCE, with 4 possible sources: markers proposed by Salipante et al. [1], used in the Idylla® assay [2], proposed by Hause et al. [3] and/or used in the revised Bethesda guideline-compliant PCR [4]. The 29 markers indicated in bold were used for the final DeltaMSI model. Genomic coordinates (start-stop) on hg19.

**2. Supplementary Figure 1**

**
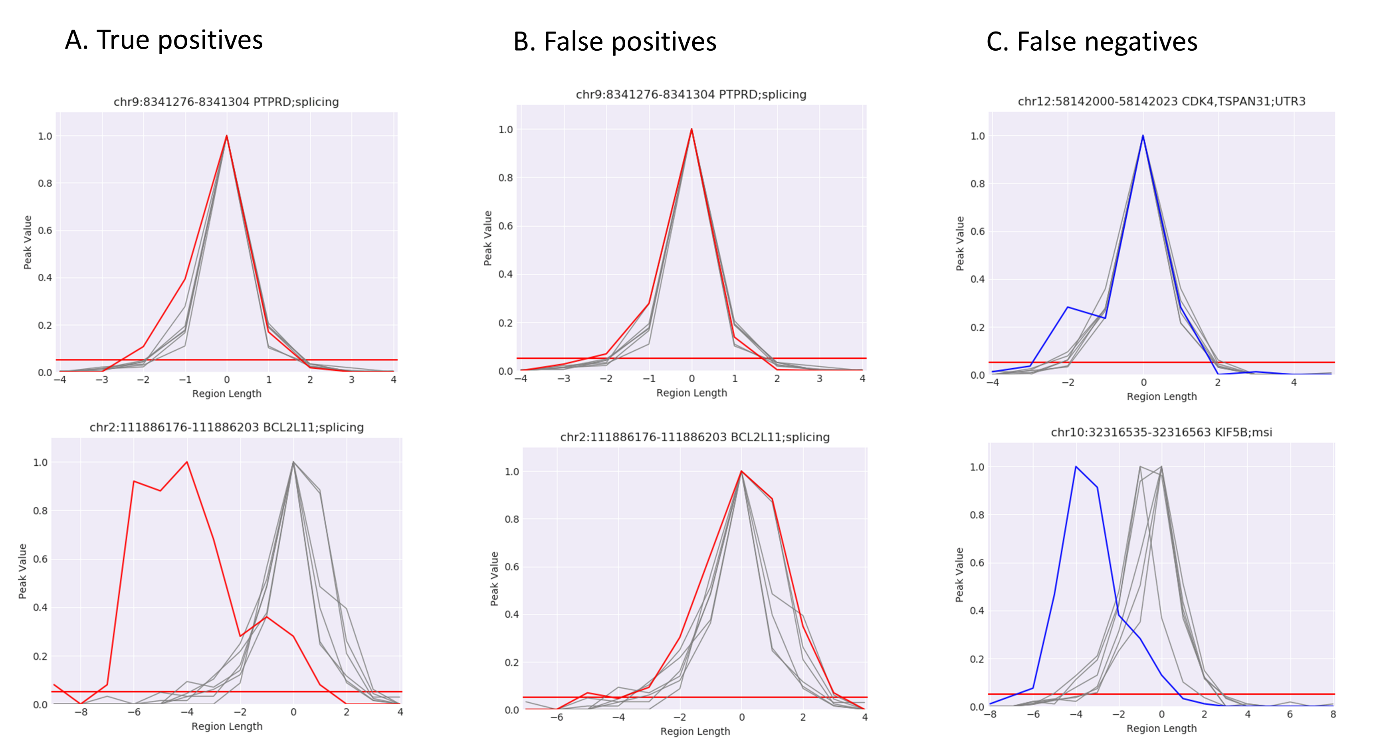
**

**Supplementary figure 1 (Fig. S1):** **inaccuracies of microsatellite stability scoring at locus level using single parameter-analysis.** The plots show indel length distributions obtained by mSINGS as described [5]. Each plot shows the expected microsatellite distribution in a reference set of microsatellite stable control samples (gray lines) for the indicated locus and the test sample indel distribution (red if scored as abnormal; blue if scored as normal). mSINGS counts the number of integrated indel peaks (>5% of total locus reads assigned to that indel length) and scores any locus as instable/positive if the number of indel length peaks is statistically greater than in control samples. Panel A shows 2 loci correctly (versus expert operator-dependent visual interpretation) called as instable (true positive). Panel B shows two examples of sample loci with visually identical distribution but falsely scored as unstable due to rigid thresholding during peak integration. Panel C shows two examples of loci falsely called stable: these distributions show minimal left shift (upper) or marked left shift (lower) of indel distribution but are not recognized by mSINGS because total number of integrated peaks remains identical as in controls.

**3. Selection of a single binary threshold for clinical use based on ROC analysis**

**Supplementary Table 2:** the selection of the optimal binary threshold is illustrated here for the SVC model of DeltaMSI. Heading of the table lists the model (SVC), the binary target (normal/abnormal IHC result) and the sample size (N=215), with a prevalence of abnormal IHC result (dMMR status) of 23.26%. The lower panel of the table lists from left to right the threshold (SVC probability score) with the associated sensitivity (95%CI), specificity (95%CI) and positive likelihood ratio (LR) to confirm presence of abnormal IHC, and negative likelihood ratio (LR) to rule out abnormal IHC. From top to bottom these diagnostic performance indicators are shows for ascending values of the threshold. A single threshold (>0.26) could be defined associated with 90% sensitivity at 98% specificity and high positive likelihood ratio (+LR) of 74. Introduction of a lower threshold for exclusion of MSI/dMMR was not useful since negative LR did not manifestly decrease at lower thresholds.

| Variable | SVC |
| --- | --- |
| Classification variable | IHC_binary |

| Sample size | 215 |
| --- | --- |
| Positive group ^a^ | 50 (23.26%) |
| Negative group ^b^ | 165 (76.74%) |

^a^ IHC_binary = 1
^b^ IHC_binary = 0

| Criterion | Sensitivity | 95% CI | Specificity | 95% CI | +LR | -LR |
| --- | --- | --- | --- | --- | --- | --- |
| ≥0 | 100.00 | 92.9 - 100.0 | 0.00 | 0.0 - 2.2 | 1.00 |  |
| >0 | 98.00 | 89.4 - 99.9 | 41.82 | 34.2 - 49.7 | 1.68 | 0.048 |
| >0.052631579 | 94.00 | 83.5 - 98.7 | 55.76 | 47.8 - 63.5 | 2.12 | 0.11 |
| >0.055555556 | 94.00 | 83.5 - 98.7 | 60.61 | 52.7 - 68.1 | 2.39 | 0.099 |
| >0.058823529 | 94.00 | 83.5 - 98.7 | 61.82 | 53.9 - 69.3 | 2.46 | 0.097 |
| >0.066666667 | 94.00 | 83.5 - 98.7 | 63.03 | 55.2 - 70.4 | 2.54 | 0.095 |
| >0.105263158 | 90.00 | 78.2 - 96.7 | 73.94 | 66.5 - 80.5 | 3.45 | 0.14 |
| >0.111111111 | 90.00 | 78.2 - 96.7 | 75.15 | 67.8 - 81.5 | 3.62 | 0.13 |
| >0.117647059 | 90.00 | 78.2 - 96.7 | 76.36 | 69.1 - 82.6 | 3.81 | 0.13 |
| >0.133333333 | 90.00 | 78.2 - 96.7 | 76.97 | 69.8 - 83.2 | 3.91 | 0.13 |
| >0.153846154 | 90.00 | 78.2 - 96.7 | 77.58 | 70.4 - 83.7 | 4.01 | 0.13 |
| >0.157894737 | 90.00 | 78.2 - 96.7 | 87.88 | 81.9 - 92.4 | 7.42 | 0.11 |
| >0.166666667 | 90.00 | 78.2 - 96.7 | 88.48 | 82.6 - 92.9 | 7.82 | 0.11 |
| >0.176470588 | 90.00 | 78.2 - 96.7 | 89.70 | 84.0 - 93.9 | 8.74 | 0.11 |
| >0.181818182 | 90.00 | 78.2 - 96.7 | 90.30 | 84.7 - 94.4 | 9.28 | 0.11 |
| >0.210526316 | 90.00 | 78.2 - 96.7 | 94.55 | 89.9 - 97.5 | 16.50 | 0.11 |
| >0.222222222 | 90.00 | 78.2 - 96.7 | 95.76 | 91.5 - 98.3 | 21.21 | 0.10 |
| >0.235294118 | 90.00 | 78.2 - 96.7 | 96.36 | 92.3 - 98.7 | 24.75 | 0.10 |
| **>0.263157895** | **90.00** | **78.2 - 96.7** | **98.79** | **95.7 - 99.9** | **74.25** | **0.10** |
| >0.315789474 | 88.00 | 75.7 - 95.5 | 100.00 | 97.8 - 100.0 |  | 0.12 |
| >0.368421053 | 84.00 | 70.9 - 92.8 | 100.00 | 97.8 - 100.0 |  | 0.16 |
| >0.388888889 | 82.00 | 68.6 - 91.4 | 100.00 | 97.8 - 100.0 |  | 0.18 |
| >0.421052632 | 78.00 | 64.0 - 88.5 | 100.00 | 97.8 - 100.0 |  | 0.22 |
| >0.473684211 | 70.00 | 55.4 - 82.1 | 100.00 | 97.8 - 100.0 |  | 0.30 |
| >0.5 | 68.00 | 53.3 - 80.5 | 100.00 | 97.8 - 100.0 |  | 0.32 |
| >0.526315789 | 64.00 | 49.2 - 77.1 | 100.00 | 97.8 - 100.0 |  | 0.36 |
| >0.578947368 | 60.00 | 45.2 - 73.6 | 100.00 | 97.8 - 100.0 |  | 0.40 |
| >0.631578947 | 56.00 | 41.3 - 70.0 | 100.00 | 97.8 - 100.0 |  | 0.44 |
| >0.647058824 | 54.00 | 39.3 - 68.2 | 100.00 | 97.8 - 100.0 |  | 0.46 |
| >0.684210526 | 50.00 | 35.5 - 64.5 | 100.00 | 97.8 - 100.0 |  | 0.50 |
| >0.6875 | 48.00 | 33.7 - 62.6 | 100.00 | 97.8 - 100.0 |  | 0.52 |
| >0.736842105 | 40.00 | 26.4 - 54.8 | 100.00 | 97.8 - 100.0 |  | 0.60 |
| >0.789473684 | 36.00 | 22.9 - 50.8 | 100.00 | 97.8 - 100.0 |  | 0.64 |
| >0.833333333 | 32.00 | 19.5 - 46.7 | 100.00 | 97.8 - 100.0 |  | 0.68 |
| >0.842105263 | 22.00 | 11.5 - 36.0 | 100.00 | 97.8 - 100.0 |  | 0.78 |
| >0.888888889 | 18.00 | 8.6 - 31.4 | 100.00 | 97.8 - 100.0 |  | 0.82 |
| >0.894736842 | 12.00 | 4.5 - 24.3 | 100.00 | 97.8 - 100.0 |  | 0.88 |
| >0.9375 | 10.00 | 3.3 - 21.8 | 100.00 | 97.8 - 100.0 |  | 0.90 |
| >0.947368421 | 6.00 | 1.3 - 16.5 | 100.00 | 97.8 - 100.0 |  | 0.94 |
| >1 | 0.00 | 0.0 - 7.1 | 100.00 | 97.8 - 100.0 |  | 1.00 |

**References**

1. Salipante SJ, Scroggins SM, Hampel HL, Turner EH, Pritchard CC: **Microsatellite instability detection by next generation sequencing**. *Clin Chem* 2014, **60**(9):1192-1199.

2. Samaison L, Grall M, Staroz F, Uguen A: **Microsatellite instability diagnosis using the fully automated Idylla platform: feasibility study of an in-house rapid molecular testing ancillary to immunohistochemistry in pathology laboratories**. *J Clin Pathol* 2019, **72**(12):830-835.

3. Hause RJ, Pritchard CC, Shendure J, Salipante SJ: **Classification and characterization of microsatellite instability across 18 cancer types**. *Nat Med* 2016, **22**(11):1342-1350.

4. Umar A, Boland CR, Terdiman JP, Syngal S, de la Chapelle A, Ruschoff J, Fishel R, Lindor NM, Burgart LJ, Hamelin R *et al*: **Revised Bethesda Guidelines for hereditary nonpolyposis colorectal cancer (Lynch syndrome) and microsatellite instability**. *J Natl Cancer Inst* 2004, **96**(4):261-268.

5. Dedeurwaerdere F, Claes KB, Van Dorpe J, Rottiers I, Van der Meulen J, Breyne J, Swaerts K, Martens G: **Comparison of microsatellite instability detection by immunohistochemistry and molecular techniques in colorectal and endometrial cancer**. *Scientific reports* 2021, **11**(1):12880.
